# Statin Therapy in Very Old Patients: Lights and Shadows

**DOI:** 10.3389/fcvm.2021.779044

**Published:** 2021-11-29

**Authors:** Lidia Cobos-Palacios, Jaime Sanz-Cánovas, Mónica Muñoz-Ubeda, María Dolores Lopez-Carmona, Luis Miguel Perez-Belmonte, Almudena Lopez-Sampalo, Ricardo Gomez-Huelgas, Maria Rosa Bernal-Lopez

**Affiliations:** ^1^Department of Internal Medicine, Regional University Hospital of Málaga-Instituto de Investigación Biomédica de Málaga (IBIMA), University of Málaga, Málaga, Spain; ^2^CIBER Fisiopatología de la Obesidad y la Nutrición, Carlos III Health Institute, Madrid, Spain

**Keywords:** statins, elderly, cardiovascular prevention, frailty, review

## Abstract

Atherosclerotic cardiovascular diseases (ASCVD) are the leading cause of death worldwide. High levels of total cholesterol—and of low-density lipoprotein cholesterol in particular—are one of the main risk factors associated with ASCVD. Statins are first-line treatment for hypercholesterolemia and have been proven to reduce major vascular events in adults with and without underlying ASCVD. Findings in the literature show that statins reduce coronary and cerebrovascular morbidity and mortality in middle-aged people, but their benefits in older adults are not as well-established, especially in primary prevention. Furthermore, many particularities must be considered regarding their use in old subjects, such as age-related changes in pharmacokinetics and pharmacodynamics, comorbidities, polypharmacy, and frailty, which decrease the safety and efficacy of statins in this population. Myopathy and a possible higher risk of falling along with cognitive decline are classic concerns for physicians when considering statin use in the very old. Additionally, some studies suggest that the relative risk for coronary events and cardiovascular mortality associated with high levels of cholesterol decreases after age 70, making the role of statins unclear. On the other hand, ASCVD are one of the most important causes of disability in old subjects, so cardiovascular prevention is of particular interest in this population in order to preserve functional status. This review aims to gather the current available evidence on the efficacy and safety of statin use in very old patients in both primary and secondary prevention.

## Introduction

According to the Global Burden of Disease study, atherosclerotic cardiovascular diseases (ASCVD) are the leading cause of death worldwide and one of the most common causes of morbidity in industrialized countries (1). Their prevention is one of the most important clinical challenges of the twenty first century. Hypercholesterolemia—and high levels of low-density lipoprotein cholesterol (LDL-c) in particular—are independent risk factors for ASCVD and one of the targets when it comes to preventing cardiovascular diseases.

Statins are first-line drugs for lipid-lowering therapy and an essential part of cardiovascular prevention. In recent decades, the benefit of statin use in the younger population has been demonstrated. There is an increase in the use of statins in adults ≥40 years of age given that they have been proven to reduce cardiovascular events, even in people with low risk of vascular disease (2). However, statins are not free of adverse effects in the general population. Statin-associated muscle symptoms are the most common statin toxicity or intolerance. In addition, other important side effects have to be considered, including hepatotoxicity, new-onset diabetes, nephrotoxicity, and neurological effects (3, 4).

The proportion of the population over 75 years of age is increasing in industrialized countries, where ASCVD are more common. However, there are no strong recommendations available on statin therapy in this population given that data from clinical trials are limited and controversial, especially in regard to primary prevention. This review aims to gather the current available evidence on the efficacy and safety of statin use in very old patients in both primary and secondary prevention.

## Atherosclerotic Cardiovascular Diseases and Hypercholesterolemia in Very Old Patients

According to demographic predictions, subjects older than 75 years of age will constitute more than 10% of the population by 2050 (5) and people over 85 years of age will be the age group with the highest population growth (6). Aging is one of the most important risk factors for ASCVD (7). In 2019, approximately 18.6 million deaths worldwide were attributed to ASCVD (8) and old patients are more vulnerable to fatal ASCVD. Subjects over 75 years have a cardiovascular mortality rate almost three times higher than the younger population (9). Furthermore, over 80% people who die due to ASCVD are older than 65 years (10). As ASCVD is one of the leading causes of morbidity and functional disability in old subjects, cardiovascular prevention in this population will be a major public health problem in the near future.

In industrialized countries, there is a high prevalence of hypercholesterolemia in old subjects. According to some epidemiological data, dyslipidemia is present in 25% of men and 42% of women older than 65 years (11). In Spain, 56% of men and 69% of women over 65 years have serum LDL-c levels >130 mg/dL (12). Despite these figures, there is a dearth of clinical trials specifically designed to establish therapeutic goals for the treatment of hypercholesterolemia in old subjects.

Some particularities must be taken into account when assessing the need to start lipid-lowering treatment in very old patients. One is age-related changes in cholesterol metabolism. Data from epidemiological studies have shown that serum total cholesterol and LDL-c levels increase until subjects reach 60–70 years due to decreased LDL-c catabolism and then, after this age, levels stabilize or decline slightly (13). Some studies suggest that the relative risk for coronary events and cardiovascular mortality associated with high serum LDL-c levels decreases after age 70 (14). Furthermore, according to epidemiological data, low serum total cholesterol levels could be associated with increased all-cause mortality in the old. In patients 80 years of age or older, higher total cholesterol levels are related to higher life expectancy (15).

## Cardiovascular Risk Assessment in Very Old Patients

The 2019 European Guidelines on Cardiovascular Disease Prevention recommend estimating total cardiovascular risk based on the SCORE (Systematic COronary Risk Evaluation) equation in adults between 40 and 65 years. SCORE, introduced in the 2003 European Society of Cardiology guidelines, predicts the 10-year risk of dying from ASCVD. The predictors included in SCORE are age, sex, smoking, systolic blood pressure, and total cholesterol (16). The 2019 American College of Cardiology (ACC) and American Heart Association (AHA) Guidelines on the Primary Prevention of Cardiovascular Disease also recommend estimating total cardiovascular risk in order to establish preventive therapy. However, their calculator has an upper age limit of 79 years (17). Likewise, Framingham data, on which the Canadian Cardiovascular Society guidelines are based, only apply to people under 75 years of age (18).

Age is one of the most important factors used in current risk score equations and thus, most of people over 75 years of age will be classified as high risk (19), though some new risk scales for very old patients have recently been developed (20). In any case, cardiovascular risk assessment in old patients requires a comprehensive geriatric evaluation that assesses functional ability, multimorbidity, polypharmacy, frailty, cognitive status, and mental health. One of the most representative characteristics of the old population is its wide range of clinical, functional, and cognitive statuses, which determine life expectancy and quality of life. This makes it necessary for physicians to individualize cardiovascular risk assessment in very old patients (21). Functional status must be evaluated when estimating cardiovascular risk, since it has more weight in estimating mortality in old individuals than traditional cardiovascular risk factors (22). In addition, patient preferences have to be considered and there must be shared decision-making that takes into account potential benefits and harms.

## Statin Use in the Old: Prescribing Trends and Side Effects

Statins, which are inhibitors of 3-hydroxy-3-methylglutaryl coenzyme A reductase, are first-line drugs for hypercholesterolemia as they reduce the synthesis of cholesterol, especially LDL-c. In the last 20 years, statins have been proven to reduce major vascular events in adults with and without underlying ASCVD, (23) but there is a lack of strong evidence on the efficacy and safety of statin use in very old adults, especially in those with frailty and multimorbidity (24). Older adults, and very old adults in particular, are classically underrepresented in clinical trials.

Despite the lack of solid evidence on the use of statins in this age group, the prescribing of statins in patients older than 75 years has experienced a more than 3-fold increase in recent decades (25). Indeed, almost one in three patients older than 80 years, is on statin therapy in industrialized countries and many of them do not have previous ASCVD (26). On the other hand, there is a trend toward discontinuing the use of statins in people older than 75 years with previous ASCVD (27). Some authors have named it the “treatment-risk paradox”: prescribing statins tends to decrease when cardiovascular risk and the risk of cardiovascular death increase. A study which included 271,504 old patients with an indication for lipid-lowering therapy (established ASCVD and/or type 2 diabetes) found that only 19.1% were on statin therapy. Age and cardiovascular risk were inversely related to the probability of receiving statins (28). This “treatment-risk paradox” in the old may be based on a lack of real cardiovascular risk perception by physicians in the old (29), an increase in multimorbidities and polypharmacy which could affect statin treatment compliance (30), and physicians' concerns about the efficacy and safety of the use of statins in this population. For these reasons, older adults are frequently under-treated in terms of cardiovascular prevention and especially when using statin therapy, although recent studies have shown that lipid-lowering treatment in this age group is as safe as in younger population (31). Cardiovascular events increase with age and hypercholesterolemia is one of the main risk factors so lipid-lowering treatment in old adults at risk is of special interest.

As reported, many peculiarities must be considered when using statin therapy in the old, including multimorbidity and polypharmacy, which can affect treatment efficacy and safety (32). Drug-drug interactions are a common problem in patients with polypharmacy. Statins metabolized through cytochrome p450 (lovastatin, simvastatin, and atorvastatin) can have multiple interactions with drugs that inhibit this pathway, such as diltiazem, verapamil, amiodarone (common drugs in patients with ASCVD), and some others such as macrolides and azole antifungals. This interaction increases the level of statins and can cause myalgia, one of the most common side effects of statin therapy (33). In very old patients, especially those with polypharmacy, fluvastatin, pravastatin, and rosuvastatin are better options because they have fewer drug interactions and a better safety profile. These observations should be taken into account when prescribing statins, but they do not necessarily need to entail deprescribing in all old patients with polypharmacy. A recent population-based cohort study of 29,047 patients aged 65 or older from the Lombardy region of Italy examined the effects of statin therapy deprescribing in old patients with polypharmacy. Discontinuing therapy was associated with a significantly increased probability of hospital admission for any cardiovascular event and all-cause mortality (34).

In addition to optimizing the type of statin, a lower dose is generally recommended for very old patients. This is based on data from some clinical trials that suggest that the statin dose can be related to adverse effects. In the TNT (Treating to New Targets) trial, participants over 65 years had a higher rate of adverse effects with 80 mg of atorvastatin than with 10 mg (8.3% vs. 5.2%) and higher treatment withdrawal (4.4 and 2.2%, respectively). Hepatic enzyme elevation was also higher among patients who received 80 mg of atorvastatin (35).

Myopathy and a possible increase in falls are one physicians' classic concerns when statin therapy is used in very old population. Unlike other events, statin-related myalgias have been widely studied in this population. A systematic review and meta-analysis by Iwere et al. examined myopathy and statin use in adults over 65 years. No differences were found in myalgias between the statin group and those not on treatment (odds ratio [OR] = 1.03; 95% confidence interval [CI] 0.9–1–17) (36). Data from the PALM (Provider Assessment of Lipid Management) registry showed that patients over 75 years reported a lower rate of myalgias compared to those younger than 75 years (27.3% vs. 33.3%) (37). Similarly, in another study, patients older than 75 years treated with statins plus ezetimibe reported similar rates of adverse events as younger patients (38). These findings seem to indicate that statin-related myalgias are non-age-related side effects.

Another classic concern about statin use in this population is treatment-related cognitive decline, which has been a point of interest since the United States Food and Drug Administration (FDA) warned about a possible link between them in 2012. A 2015 systematic review and meta-analysis (14 randomized controlled trials, n=27,643) by Ott et al. did not show a relationship between cognitive decline and statin therapy (39). An observational study conducted in Australia in 2019 evaluated cognitive status and brain volume in people 70 to 90 years of age with and without statin therapy. There were no differences in cognitive decline and brain volume between the two groups (40). A recent study conducted on healthy old adults from the ASPREE (Effects of Aspirin on All-Cause Mortality in the Healthy Old) trial found no association between statin therapy and incident dementia, mild cognitive impairment, or cognitive decline in patients over 65 years of age (41). The available evidence does not seem to show an association between statin use and cognitive decline, but with the important caveat that most of the studies have been conducted on participants younger than 75 years.

A review that encompasses the recommendations of 18 sets of international guidelines on statin discontinuation among older adults has recently been published. Many of the international guidelines included in the review recommend discontinuation of statin use when there is intolerance, which includes effects such as muscle symptoms (including rhabdomyolysis), liver toxicity, or contraindications. All these international guidelines are applicable to older adults (42).

As life expectancy increases, it is critically important to establish clear recommendations on the use of statin therapy in very old patients in order to ensure a healthy aging population.

## Other Lipid-Lowering Treatments Among Old Individuals

As in the young population, there are other therapeutic alternatives apart from statins for lipid-lowering treatment among old individuals. Several studies propose dietary modification as an effective alternative to lipid-lowering drugs (43, 44). Ezetimibe, bile acid sequestrants, fibrates, nicotinic acid, and acipimox must be used for a long time in order to achieve vascular benefits. Therefore, for short-term use, the risk of adverse effects outweighs the potential benefits in senescence (45). However, there are clinical trials that support the use of other drugs. The EWTOPIA 75 trial showed that ezetimibe prevents cardiovascular events in primary prevention in patients older than 75 years (46). Another study shows the efficacy of niacin and fibrates in reducing triglyceride levels and increasing high-density lipoprotein (HDL) cholesterol levels; they are a safe and feasible combination in very old patients (47).

## Evidence on the Use of Statins For Cardiovascular Prevention in Very Old Patients

### Primary Prevention

Data from studies show that statins reduce coronary and cerebrovascular mortality and morbidity in middle-aged people. However, their benefits in older adults are not as well-established, especially in regard to primary prevention, due to a dearth of randomized studies performed in people over 75 years of age (48).

Most of the current available evidence on the use of statins for primary cardiovascular prevention in patients older than 75 years come from subgroup analyses of larger studies, which are summarized in Table 1. The PROSPER (Prospective Study of Pravastatin in the Old at Risk) trial (n = 5,084) was the first study specifically designed to evaluate the efficacy of statins in the reduction of major vascular events in both primary and secondary prevention in old patients. PROSPER examined the efficacy of pravastatin vs. placebo in people aged 70–82 years (median age 75) from the Netherlands, Scotland, and Ireland. The primary endpoint was a composite of coronary death, non-fatal MI, and fatal and non-fatal stroke. After a mean follow-up period of 3.2 years, there was a significant reduction in the primary endpoint in the pravastatin group (hazard ratio [HR] = 0.85; 95% CI 0.74–0.97), but not in patients in primary prevention (HR = 0.94; 95% CI 0.77–1.15) (49).

**Table 1:** Available studies on statin use for primary and secondary cardiovascular prevention in older subjects.

**Study**	**Year**	**Prevention**	**Population**	**Number of older subjects** **(Age range)**	**Intervention**	**Follow-up** **(years)**	**Results**
PROSPER (49)	2002	PrimarySecondary	5,804 patients with vascular disease or risk factors and a TC level of 154 mg/dL	5,084 (70–82 years)	Pravastatin 40 mg vs. placebo	3.2	Reduction in the primary endpoint (death due to CHD, non-fatal MI, or stroke) with pravastatin compared to placebo (HR 0.85; 95% CI 0.74–0.97), but not in primary prevention (HR 0.94; 95% CI 0.77–1.15)
JUPITER (50)	2010	Primary	17,802 patients without CVD, LDL-c <130 mg/dL, and CRP (> 2 mg/L)	5,695 (>70 years)	Rosuvastatin 20 mg vs. placebo	5	Reduction in the incidence of major cardiovascular events with rosuvastatin compared to placebo (HR 0.61, 95% CI 0.46–0.82). Similar rate of any serious adverse event in this age range (HR 1.05, 95% CI 0.93–1.17)
HOPE-3 trial (51)	2016	Primary	12,705 (55–72 years) patients without ASCVD	Women aged ≥ 65 years or women ≥ 60 years with at least two additional risk factors and men aged ≥ 55 years	Rosuvastatin 10 mg vs. placebo	5.6	Rosuvastatin reduced the risk of cardiovascular events (death from cardiovascular causes, non-fatal MI, or non-fatal stroke) (HR 0.76, 95% CI 0.64–0.91), with a similar effect seen in the subgroup of patients >65 years (mean age 71 years)
ALLHAT-LLT (52)	2017	Primary	2,867 patients without ASCVD	2,867 (≥ 65 years)	Pravastatin 40 mg vs. placebo	6	Pravastatin does not benefit older adults with moderate hyperlipidemia and hypertension and causes a non-significant increase in all-cause mortality among adults ≥75 years. HR for all-cause mortality in the pravastatin group were 1.18 (95% CI 0.97–1.42), 1.08 (95% CI 0.85–1.37), and 1.34 (95% CI 0.98–1.84) for all adults ≥65 years, 65–74 years, and ≥75 years, respectively (*p* > 0.05 in all cases)
Retrospective cohort study (53)	2018	Primary	46,864 people without ASCVD	46,864 (≥75 years)	Statin treatment: simvastatin, pravastatin, lovastatin, fluvastatin, rosuvastatin, atorvastatin	5.6	Population without T2DM:- 75–84 years: HR 0.94 (95% CI 0.86–1.04) for ASCVD and 0.98 (95% CI 0.91–1.05) for all-cause mortality - ≥85 years: HR 0.76 (95% CI 0.82–1.06) for ASCVD and 0.97 (95% CI 0.90–1.05) for all-cause mortality Population with T2DM: - 75–84 years: HR 0.94 (95% CI 0.65–0.89) for ASCVD and 0.84 (95% CI 0.75–0.94) for all-cause mortality - ≥85 years: HR 0.82 (95% CI 0.53–1.26) for ASCVD and 1.05 (95% CI 0.86–1.28) for all-cause mortality In participants with diabetes, statins showed a protective effect, but this effect was reduced beyond the age of 85 years and disappeared in non-agenarians
Retrospective cohort study (54)	2020	Primary	57,178 patients without ASCVD	57,178 (≥75 years)	Statin treatment: atorvastatin, cerivastatin, fluvastatin, lovastatin, pitavastatin, pravastatin, rosuvastatin, simvastatin	6.8 ± 3.9	HR 0.75 (95% CI 0.74–0.76) for all-cause mortality, HR 0.80 (95% CI 0.78–0.81) for CV mortality, and HR 0.92 (95% CI 0.91–0.94) for ASCVD events
Systematic review of observational studies (55)	2021	Primary	815,667 patients without ASCVD	815,667 patients (≥ 65 years)	Statin therapy	4.7–24.0	Statin therapy was associated with a significantly lower risk of all-cause mortality [HR: 0.86 (95% CI 0.79 to 0.93)], CVD death [HR: 0.80 (95% CI 0.78 to 0.81)], and stroke [HR: 0.85 (95% CI 0.76 to 0.94)] and a non-significant association with risk of MI [HR 0.74 (95% CI 0.53 to 1.02)]. The beneficial association of statins with the risk of all-cause mortality remained significant even among elderly individuals [> 75 years old; HR 0.88 (95% CI 0.81 to 0.96)]
4S (59)	1994	Secondary	4,444 subjects with ASCHD and hypercholesterolemia	1,021 (65–70 years)	Simvastatin 20–40 mg vs. placebo	5.4	Simvastatin benefits: RR 0.70 (95% CI 0.58–0.85) for mortality RR 0.66 (95% CI 0.59–0.75) for major coronary events A 37% reduction in the risk of undergoing myocardial revascularization procedures (*p* <0.00001)
HPS (60)	2002	Secondary	20,536 subjects with ASCVD, DM, or HT and TC level ≥135 mg/dL	5,806 (70–80 years)	Simvastatin 40 mg vs. placebo	5	Simvastatin significantly reduces coronary events (27%), stroke (25%), revascularization and cardiovascular events (24%), CV death (17%), and all-cause mortality (13%).Also, occurrence of a first major cardiovascular event declined in all age groups: - 24% in subjects <65 years groups: -23% in subjects 65–69 years groups: -18% in subjects 70–80 years
SAGE (61)	2007	Secondary	893 outpatients with CAD	893 patients (65–85 years)	Atorvastatin 80 mg vs. pravastatin 40 mg	1	Both treatments significantly reduced total duration of ischemia at 3 and 12 months Greater reduction of LDL-c levels was observed with atorvastatin than pravastatin, and also a trend to fewer major cardiovascular events (HR 0.71, 95% CI 0.46–1.09), and a significant reduction in all-cause mortality (HR 0.33, 95% CI 0.13–0.83)
Meta-analysis. Cholesterol Treatment Trialists' Collaboration (62)	2019	PrimarySecondary	186,804 patients with/without ASCVD	14,483 patients (≥75 years)	Statin therapy vs. placebo	4.8	In people ≥75 years, each 1 mmol/L LDL-c reduction was associated with decreased risk for major vascular events (RR 0.82, 95% CI 0.70–0.95) and major coronary events (RR 0.82, 95% CI 0.70–0.96) A reduction in major vascular events in people with previous ASCVD (RR 0.85, 95% CI 0.73–0.98) was observed, but there was no significant reduction in people without previous ASCVD (RR 0.92, 95% CI 0.73–1.16)
Systematic review (63)	2019	PrimarySecondary	60,194 patients with ASCVD, DM	60,194 patients (≥65 years)	Statin therapy vs. placebo	1	For primary prevention, statins reduced the risk of CAD (RR 0.79, 95% CI 0.68–0.91) and MI (RR: 0.45, 95% CI 0.31–0.66), but not all-cause or cardiovascular mortality or stroke. There was no significant interaction between diabetes status and the intervention's effect For secondary prevention, statins reduced all-cause mortality (RR 0.80, 95% CI 0.73–0.89), cardiovascular mortality (RR 0.68, 95% CI 0.58–0.79), CAD (RR 0.68, 95% CI 0.61–0.77), MI (RR 0.68, 95% CI 0.59–0.79), and revascularization (RR 0.68, 95% CI 0.61–0.77)

*TC, Total Cholesterol; CHD, coronary heart disease; MI, myocardial infarction; HR, Hazard ratio; CI, Confidence Interval; CVD, Cardiovascular Disease; LDL-c, Low-Density Lipoprotein cholesterol; CRP, C-Reactive Protein; HDL-c, High-Density Lipoprotein cholesterol; CV, Cardiovascular; RR, Relative Risk; DM, Diabetes Mellitus; HT, Hypertension; CAD, Coronary Artery Disease*.

JUPITER (Justification for the Use of Statins in Prevention: an Intervention Trial Evaluating Rosuvastatin) evaluated the efficacy of rosuvastatin in the prevention of major vascular events in people with LDL-c <130 mg/dL and elevated high-sensitive C-reactive protein (> 2 mg/L). This trial enrolled a large number of old patients: 5,695 of the participants were 70 years or older. This subgroup of individuals over 70 years of age accounted for 49% of the primary endpoint events (*n* = 194) and rosuvastatin significantly reduced vascular events in this subgroup (HR = 0.61; 95% CI 0.46–0.82). In addition, the absolute risk reduction was higher in the older group compared to younger participants (50).

The HOPE-3 trial (Heart Outcomes Prevention Evaluation) examined the efficacy of 10 mg/day of rosuvastatin vs. placebo in the reduction of cardiovascular risk in 12,705 subjects without ASCVD in 21 countries. In the subgroup of people over 70 years, statin therapy did not reduce the cardiovascular risk after 5 years of treatment (51).

A subanalysis of the ALLHAT-LLT (Antihypertensive and Lipid-lowering Treatment to Prevent Heart Attack Trial Lipid-Lowering Treatment Trial) study concluded that pravastatin did not reduce cardiovascular events in very old adults on primary prevention (52).

A population-based cohort study evaluated the reduction of ASCVD and all-cause mortality in very old Spanish adults without previous ASCVD (53). A total of 46,864 people aged 75 years and older (mean age 77 years, median follow-up time 5.6 years) were enrolled from a national database. Subjects with previous ASCVD, type 1 diabetes, dementia, cancer, previous lipid-lowering treatment, and those who were in residential care, on dialysis, or had received an organ transplant were excluded. Subjects were classified by age (75–84 years and 85 years and older), diabetes diagnosis (diagnosis with type 2 diabetes or not), and statin therapy (non-user or new user). In the 75–84 years of age group, the risk of ASCVD and all-cause mortality in people with type 2 diabetes was lower than in those who receive statin therapy compared to those who did not receive statin therapy (HR=0.76; 95% CI 0.65–0.89 for ASCVD and HR = 0.84; 95% CI 0.75–0.94 for all-cause mortality). In individuals 75–84 years of age without type 2 diabetes, there was no difference in risk of ASCVD or all-cause mortality. Similarly, in the 85 years and older age group, there was no difference in the risk of ASCVD or all-cause mortality regardless of diabetes status.

Another recent population-based cohort study conducted in the US Veterans Health Administration population that included 57,178 subjects older than 75 years without ASCVD for a mean follow-up period of 6.8 years. Use of statins was associated with a 25% reduction in all-cause mortality and a 20% reduction in cardiovascular mortality (54). It should be noted that despite the large number of enrolled patients, the latter two reports are retrospective population-based studies with a weak evidence level.

The latest systematic review and meta-analysis of observational studies expands upon the current evidence on statin use for primary prevention among old subjects. This recent meta-analysis included nine cohorts and one case control study with 815,667 patients without ASCVD. Statin therapy was associated with a significantly lower risk of all-cause mortality (HR: 0.86 [95% CI 0.79–0.93]), ASCVD death (HR: 0.80 [95% CI 0.78–0.81]), and stroke (HR: 0.85 [95% CI 0.76–0.94]) and a non-significant association was found with risk of MI (HR 0.74 [95% CI 0.53–1.02]). The beneficial association of statins on the risk of all-cause mortality remained significant even among older individuals (>75 years old; HR 0.88 [95% CI 0.81–0.96]) (55).

More clinical trials in subjects older than 75 years are needed, given the dearth of solid data on the use of statins for primary prevention in this population. STAREE (Statins for Reducing Events in the Old) is an ongoing trial that aims to evaluate the efficacy of 40 mg/day of atorvastatin vs. placebo in 18,000 subjects over 70 years without prior ASCVD. Its results are expected to be made known in 2022 (56). PREVENTABLE (PRagmatic EValuation of evENTs And Benefits of Lipid-lowering in oldEr Adults) is the latest ongoing trial evaluating the efficacy of statins in very old patients without ASCVD. This large study, initiated in September 2020, aims to demonstrate the benefit of atorvastatin 40 mg/day in reducing the primary endpoint of death, dementia, and persistent disability and a secondary composite endpoint of mild cognitive impairment and vascular events. No data are available yet as PREVENTABLE will be in the recruitment phase until June 2026 (57).

The latest international guidelines do not provide general recommendations on the use of statin therapy for primary prevention in individuals 75 years of age or older. In the 2018 American Heart Association (AHA) and American College of Cardiology (ACC)—Multi-Society Cholesterol Guidelines, there is scarce data and unclear evidence for a strong, risk-based general statin therapy recommendation in people older than 75 years without previous cardiovascular events (58). In addition, both European Society of Cardiology (ESC) and AHA/ACC guidelines recommend special consideration when prescribing statins in very old people, especially when it comes to high doses or intensity, as they increase the risk of adverse effects without improving life expectancy and also decrease quality of life.

### Secondary Prevention

Most of the strong evidence for the use of statins in the old is derived from trials with subjects with established ASCVD but most data on people aged 75 and over come from subgroup analyses (Table 1).

4S (Scandinavian Simvastatin Survival Study) was the first randomized controlled trial to prove the efficacy of statins (simvastatin 20–40 mg/day vs. placebo) in secondary prevention (59). This study enrolled 4,444 patients with previous history of ASCVD, but only 1,021 subjects were older than 65 years and the maximum age was 70.

The HPS (Heart Protection Study) trial enrolled 20,536 subjects, 5,806 of which were 70–80 years of age, and had a mean follow-up period of 5 years. Compared to a placebo, simvastatin reduced all-cause mortality by 13% and cardiovascular mortality by 27% regardless of age. Simvastatin decreased the rate of a first major cardiovascular event by 18% in participants aged 70–80 years (60).

A smaller randomized trial entitled SAGE (Study Assessing Goals in the Old) included 893 outpatients aged 65–85 years and compared intensive statin therapy (atorvastatin 80 mg/day) vs. moderate therapy (pravastatin 40 mg/day) for secondary prevention. After 12 months, atorvastatin significantly reduced LDL-c levels and all-cause mortality and led to a non-significant trend toward fewer acute major cardiovascular events (61).

A 2019 meta-analysis of 28 randomized controlled trials examined the reduction of major vascular and coronary events in people older than 55 years on statin therapy (62). In people aged 75 years and older (n = 14,483, 8% of the total participants) who were followed-up on for a mean of 4.9 years, each 1 mmol/L reduction in LDL-c was associated with a decrease in risk of major vascular events (relative risk [RR] = 0.82; 95% CI 0.70–0.95) and major coronary events (RR = 0.82; 95% CI 0.70–0.96). Comparing people with and without previous ASCVD, there was a reduction in major vascular events in participants with previous ASCVD (RR = 0.85; 95% CI 0.73–0.98), but no significant reduction in those without previous ASCVD (RR = 0.92; 95% CI 0.73–1.16).

A 2019 systematic review that included 60,194 patients aged 65 years and older from 23 trials showed significant reductions in all-cause mortality (RR = 0.80; 95% CI 0.73–0.89), cardiovascular mortality (RR = 0.68; 95% CI 0.58–0.79), and major coronary events (RR = 0.68; 95% CI 0.61–0.77) in patients with established ASCVD on statin therapy. However, there was no significant reduction in mortality in primary prevention (63).

As the use of statins for secondary prevention in old subjects is supported by more robust and less controversial evidence, the latest international guidelines provide some general recommendations on their use. The 2019 ESC Guideline for the Management of Dyslipidaemias recommends the use of statins in older adults with established ASCVD in the same way as in younger population (class I recommendation, level of evidence A), but does not provide specific recommendations for people >75 years (64). The 2018 AHA/ACC Guideline on the Management of Blood Cholesterol considers it reasonable to continue treatment with high-intensity statins if the subject tolerates statins or to initiate moderate or high-intensity statins in patients >75 years of age with established ASCVD (Class IIa recommendation) (65).

## Conclusions

Cardiovascular risk assessment in very old patients requires a comprehensive geriatric evaluation that assesses functional capacity, cognitive status, and frailty. Multimorbidity and polypharmacy can affect the efficacy and safety of and compliance with statin therapy. The indication of primary cardiovascular prevention with statins in very old patients remains unclear based on the current available evidence and the clinical decision to prescribe them must be made on a case-by-case basis. For patients with established ASCVD, statin therapy is generally recommended except for those with severely impaired health, extreme frailty, or a short life expectancy.

## Author Contributions

LC-P, RG-H, and MB-L: conceptualization. LC-P, JS-C, MM-U, and AL-S: methodology. LC-P, ML-C, LP-B, and MB-L: validation. LC-P, JS-C, MM-U, and MB-L: investigation. LC-P, RG-H, and MB-L: data curation, writing—original draft preparation, and writing—review and editing. RG-H and MB-L: visualization and supervision. All authors contributed to the article and approved the submitted version.

## Funding

This work was supported by grants from the Instituto de Salud Carlos III, cofinanced by the Fondo Europeo de Desarrollo Regional-FEDER [Centros de Investigación En Red (CIBER, CB06/03/0018)]. LC-P, JS-C and AL-S were supported by Rio Hortega program (CM20/00125, CM20/00212, and CM21/00110, respectively) from the ISCIII-Madrid (Spain), cofinanced by the Fondo Europeo de Desarrollo Regional-FEDER. MM-U was supported by Consejeria de Salud, Junta de Andalucía (RH-0100-2020). MB-L was supported by Miguel Servet Type II program (CPII/00014) from the ISCIII-Madrid (Spain), cofinanced by the Fondo Europeo de Desarrollo Regional-FEDER and Nicolas Monardes program (C1-0005-2020), supported by Consejeria de Salud, Junta de Andalucía.

## Conflict of Interest

The authors declare that the research was conducted in the absence of any commercial or financial relationships that could be construed as a potential conflict of interest.

## Publisher's Note

All claims expressed in this article are solely those of the authors and do not necessarily represent those of their affiliated organizations, or those of the publisher, the editors and the reviewers. Any product that may be evaluated in this article, or claim that may be made by its manufacturer, is not guaranteed or endorsed by the publisher.
